# Prevention of alcohol exposed pregnancies in Europe: the FAR SEAS guidelines

**DOI:** 10.1186/s12884-024-06452-9

**Published:** 2024-04-06

**Authors:** Carla Bruguera, Lidia Segura-García, Katarzyna Okulicz-Kozaryn, Claudia Gandin, Silvia Matrai, Fleur Braddick, Marta Zin-Sędek, Luiza Slodownik, Emanuele Scafato, Joan Colom

**Affiliations:** 1 Subdirectorate General of Addictions, HIV, STI and Viral Hepatitis, Public Health Agency of Catalonia (GENCAT), Barcelona, Spain; 2 Institute of Mother and Child, Warsaw, Poland; 3 National Observatory on Alcohol, National Center on Addiction and Doping, Istituto Superiore di Sanità (ISS), Rome, Italy; 4 CLÍNIC Foundation for Biomedical Research (FCRB), Barcelona, Spain; 5 Polish National Centre for Addiction Prevention (KCPU), Warsaw, Poland

**Keywords:** Prevention, Prenatal alcohol exposure, Fetal alcohol spectrum disorder (FASD), Alcohol, Pregnancy

## Abstract

**Introduction:**

Drinking during pregnancy is the leading cause of birth defects and child developmental disorders in Europe. The adverse effects of drinking during pregnancy may include physical, behavioural and cognitive problems, known collectively as fetal alcohol spectrum disorders (FASD). Evidence-based comprehensive recommendations at the European level on how to implement preventive and treatment policies to reduce alcohol-exposed pregnancies are needed. FAR SEAS, a tendered service contract (number 20,187,106) awarded by the European Commission, aimed at developing guidelines to respond to this knowledge gap.

**Methods:**

FAR SEAS recommendations were built on (1) a two-phase review of interventions, (2) an international expert consultation, and (3) a pilot study on prevention of FASD conducted in the Mazovia region of Poland. The review of interventions included nineteen electronic open access databases, several repositories of grey literature and a key informant consultation covering most European Union (EU) countries and an additional guidelines search. After triangulating sources, 94 records were collected. Experts contributed in the design of the research questions, addressing the gaps in the literature and reviewing the recommendations formulated. The Polish pilot added nuances from real world practice to the formulated recommendations, resulting in the final set of guidelines for dissemination.

**Results:**

The FAR SEAS Guidelines comprise 23 recommendations grouped into different topics areas of policies, communication strategies, screening, brief intervention and referral to treatment, treatment and social services. The recommendations highlight the need to respect women’s autonomy and avoid discrimination and stigmatization; using universal screening for women of childbearing age, including detection of other psychosocial risks (such as domestic violence); and individualized, comprehensive and multidisciplinary supportive interventions for those who require it, such as those with alcohol use disorders, including women’s partners. Policies to prevent FASD should be multicomponent, and public health communication should combine information about the risks together with self-efficacy messages to promote changes.

**Conclusions:**

The FAR SEAS guidelines are a tool to support policy-makers and service managers in implementing effective programmes to reduce prenatal alcohol exposure among general and at-risk population groups. FASD prevention has to involve comprehensive and multi-level evidence-based policies and practice, with services and activities tailored to the needs of women at differing levels of risk, and with due attention to reducing stigma.

**Supplementary Information:**

The online version contains supplementary material available at 10.1186/s12884-024-06452-9.

## Introduction

Women have traditionally consumed alcohol in lower amounts and less frequently than men. However, the gender gap has been decreasing and even disappearing in some EU countries [1]. Alcohol use is associated with breast cancer [2], alcoholic hepatitis [3], heart disease [4] and brain damage [5]. The risk for alcohol-related harms is higher when women are pregnant, as the adverse effects of drinking during pregnancy may include physical, behavioural and cognitive problems, known collectively as FASD. The global prevalence of FASD [6, 7] was estimated to be 7.7 per 1000 population (95% CI, 4.9–11.7 per 1000 population), with the European Region having the highest overall prevalence at 19.8 per 1000 population (95% CI, 14.1–28.0 per 1000 population) [8]. Overall, FASD is preventable by abstaining from drinking alcohol during pregnancy, or by effective contraception use when drinking [9].

In the context of FASD prevention policies, the EU Strategy to support Member States in reducing alcohol related harm [10] requested governments to raise awareness of the risks of drinking during pregnancy. The need for evidence-based policies to reduce alcohol related harms was also stressed. These guidelines are the response from the FAR SEAS project (Fetal Alcohol Reduction and exchange of European knowledge after the Standard European Alcohol Survey) to the absence of evidence-based guidelines at the European level on how to implement preventive interventions or treatment practices to reduce alcohol-exposed pregnancies. FAR SEAS is a tendered service contract (number 20,187,106) awarded by the European Commission, under the EU health programme.

The aim of the FAR SEAS guidelines is to provide the best available evidence to prevent and reduce alcohol consumption in women of child-bearing age, particularly in pregnant women, in an easily accessible and comprehensive format.

## Methods

### Target audience and population

The recommendations in the FAR SEAS guidelines are primarily aimed at policy makers, health service managers, health-care providers and social workers who are in contact with pregnant women and women of child-bearing age. Among health-care providers, we expect these guidelines to be useful to gynaecologists and obstetricians, physicians or primary health care professionals, midwives and nurses, and psychologists and psychiatrists working in the addiction field.

The target population to reach is women of child-bearing age, especially those who are pregnant, and includes those in vulnerable situations and disadvantaged groups or who have alcohol-related problems.

### Evidence compilation

Evidence was collected in 4 stages:

1. Literature review of peer-reviewed papers.
2. Consultation of experts.
3. Grey literature.
4. Pilot study in Poland.

Initially, a literature review was performed. Nineteen electronic open access databases were searched for relevant articles, guidelines and policies related to pregnant women and alcohol use. The databases searched were: African Journals Online (AJOL), CiNii, Cochrane, Cordis, Cuiden, Dialnet Plus, ENFISPO, InDICEs, Joanna Briggs Institute EBP Database, LILACS, Open Grey, Psicodoc, Psychology & behavioral sciences collection, PsycINFO, PubMed, SciELo, Scopus, WoS BIOSIS Preview and WoS Core Collection. The search strategy was developed by an expert bibliographer from the University of Barcelona in consultation with experts from the Hospital Clínic de Barcelona, GENCAT (Generalitat de Catalunya) and PARPA (State Agency for the Prevention of Alcohol-Related Problems).

The strategy was organized around the following three main keyword topics:

1. Pregnant women: (pregnant OR gestation OR prenatal) AND.
2. Alcohol consumption: (alcohol drinking OR alcohol consumption OR alcoholics OR alcoholism) AND.
3. Types of documents relevant for the review: (Practice Guideline OR Clinical trial OR Guideline OR Health Promotion OR Health Planning Guidelines OR Advance Directive Adherence OR Benchmarking OR Clinical Trials OR Program Development OR Program Evaluation OR Government Programs OR Preventive Health Services OR Education OR Safety Management OR Social Validity, Research OR Family Planning Services OR Healthy People Programs).

Inclusion criteria:

- From 2010.
- All geographical areas.
- Sample *N* > 6.
- All languages.
- Researches that followed equity and bioethical principles.
- Peer reviewed articles.

Exclusion criteria:

- Experimental research with animals.
- FASD diagnosis or management.

Secondly, we undertook consultations with informants from key European and international expert networks such as EUFAS (European Federation of Addiction Societies), EUFASD (European FASD Alliance), INEBRIA (International Network on Brief Interventions for Alcohol and Other Drugs), APN (Alcohol Policy Network), RARHA (Reducing Alcohol Related Harm), and 3 public health organizations – the European Monitoring Centre for Drugs and Drug Addiction (EMCDDA), World Health Organization (WHO) and the EU’s Consumer, Health, Agriculture and Food Executive Agency (CHAFEA) - who provided further documents (published articles or grey literature).

The third step was to search relevant European and international repositories of the grey literature (e.g., reports and other documents from institutions or public health organizations such as the EMCDDA, WHO, and a key network: the European Fetal Alcohol Spectrum Disorders Alliance (EUFASD)). An additional search of guidelines was performed as well as a further round of expert consultations to address gaps in the evidence. Finally, secondary sources emerging from the documents reviewed were also included.

### Evidence assessment

Data extracted were assessed to ensure a robust body of evidence:

- Randomised Control Trials (RCT) were analysed by using RoB2 [11], the revised Cochrane risk-of-bias tool for RCTs. Those studies with one or more high-risk domain were excluded.
- Systematic reviews (SRs) were assessed using AMSTAR [12]. Reviews that scored less than 4 items were excluded.
- Guidelines were evaluated by using the section *Rigour of Development criteria* of the AGREE II instrument [13]. Guidelines scoring less than 70% were excluded.

Two investigators evaluated the guidelines and a sample of RCTs and systematic reviews. A template (see Table 12 in Appendix 1) was made with the different dimensions to be evaluated, according to study design. Each researcher evaluated independently, they then met to compare scores and reach agreement by consensus. The evidence was then rated using the criteria described by the Scottish Intercollegiate Network (SIGN) ‘A guideline developer’s handbook’ [14] (see Table 1 in Appendix 1).

### Experts’ assessment

The Guidelines Development Group (see Table 2 in Appendix 1) used the evidence rating, plus further evidence on harms, benefits, values, preferences, resource use, and feasibility, to write a set of recommendations for each question of the topics.

The strength, relevance and transferability of each recommendation was assessed and determined based on the level of agreement with each recommendation by the internal reviewers and external experts (see Table 3 in Appendix 1).

Experts were selected according to their expertise in the FASD and public health fields and relevant experience. They were asked to provide feedback on the scope, aims and questions answered by these guidelines, as well as the recommendations formulated and the final version of the document.

In order to improve and explore the validity of those recommendations that presented evidence gaps, feedback wascollected via a bespoke online questionnaire, which elicited opinions from members of EUFASD (European FASD alliance) and the Kettil Brunn Society –a society for social and epidemiological research on alcohol.

### Piloting

Finally, lessons learned from the pilot study of an FASD prevention programme [15] in Mazovia (Poland), also in the context of FAR SEAS, introduced further nuances to the recommendations formulated.

The pilot project was implemented in four municipalities in Poland, recruited based on the willingness of care centres to participate, intention to address this perinatal issue, and commitment to promote the participation of multi-disciplinary professionals from different services (social, addiction, and psychology). The pilot study protocol was assessed and approved by the ethics committee of the Polish State Agency for the Prevention of Alcohol-Related Problems (formerly PARPA, now KCPU), and all participants (or their legal guardian) gave informed consent for their participation and data use in line with the EU GDPR. After the initial training based on motivational interviewing and the CHOICES programme [16], professionals recruited women of child-bearing age (pregnant and not pregnant) in their local communities, screened them for alcohol risk using standardised scales, and allocated participants (*n* = 441) to groups for low- (69%), moderate- (23%) or high-risk (7%) of alcohol-exposed pregnancy. The professionals also provided interventions tailored to the needs of each participant, ranging from short feedback, to brief intervention, to motivational interviewing sessions (to change one or both of two high-risk behaviours – risky alcohol use and/or no contraception use), to referral to other specialists. The results indicated positive changes in the key outcome variables: risky alcohol consumption, contraception use and visiting a gynaecologist, as well as in associated psychosocial risk factors (decreases in cigarette and drug use, domestic violence and depressive symptoms). Full details of the FAR SEAS Polish pilot study can be found in the resulting publication [15].

## Results

The 2626 records identified in the review were screened to exclude 2524 (682 duplicates + 1842 records that did not meet inclusion criteria) leaving 102 records included (see Fig. 1 for PRISMA chart and Table 11 in Appendix 1 for records included).

Twenty-three recommendations were formulated based on the literature reviews, several rounds of experts consultations, and the pilot study deployed in the Mazovian region of Poland. The recommendations were organised into the following 7 topics: (1) Organizational, strategic and policy changes required to properly address the needs of pregnant women and women of child-bearing age who are at risk of having, or who already have an alcohol-related problem (see Table 4 in Appendix 1); (2) Strategies and best practices for promoting and raising awareness of the risks of drinking alcohol during pregnancy (see Table 5 in Appendix 1); (3) Validated tools to screen alcohol use and maternal risk factors and further assess alcohol-related problems among pregnant women and women of child-bearing age in health and social care settings (see Table 6 in Appendix 1); (4) Preventive interventions for pregnant women and women of child-bearing age at risk of having alcohol related problems (see Table 7 in Appendix 1); (5) Treatment interventions for pregnant women and women of child-bearing age at risk of having alcohol related problems (see Table 8.1 and 8.2 in Appendix 1); (6) Social measures for pregnant women and women of child-bearing age at risk of having alcohol related problems (see Table 9 in Appendix 1); (7) Implementation, training and evaluation strategies for preventing activities (see Table 10 in Appendix 1).

Details on the formulation of specific recommendations and incorporation of lessons from the pilot can be seen in the FAR SEAS Deliverable report on this output (D13).

The final FAR SEAS guidelines were delivered in various formats addressing different beneficiaries (from an extensive deliverable report addressing technical policy makers and practitioners, to a laypersons’ guide to inform the general audience), for dissemination through scientific and public health channels throughout the final months of the FAR SEAS contract. These documents are available from the authors of this paper or FAR SEAS contract coordinators, pending publication on the EC web pages.

## Conclusions

It is well-established in scientific literature that there is no safe level of alcohol use during pregnancy [9] and that abstinence or effective contraception are the only responsible professional recommendations.

There is scarce robust evidence on which interventions are more effective to reduce alcohol exposed pregnancies. However, despite the lack of high-quality studies, recommendations need to be made in order to support women in avoiding alcohol while pregnant, and to protect the health of their future children.

These guidelines have been built upon the foundation of the WHO guidelines [17]. However, they specifically target alcohol-related issues and extend beyond clinical considerations to encompass public health perspectives.

Four main recommendations can be drawn out of the FAR SEAS project are:

- Multicomponent policies that range from primary prevention to harm reduction strategies need to be developed that consider respect for women’s autonomy and protection from discrimination.
- In order to promote changes, it is recommended that public health communication combine information about risks and self-efficacy messages.
- Universal screening is recommended (rather than targeted screening, which is open to bias and stigmatisation of certain groups) for women of childbearing age, including the detection of other psychosocial risks that can correlate with alcohol use during pregnancy, as were included in the FAR SEAS protocol, and accompanied by individualized interventions for those who require it, including their partners.
- Women with alcohol use disorders need comprehensive and multidisciplinary support also addressing psychosocial risks such as poverty or violence.

This paper has some limitations. Recommendations included here were formulated according to evidence, but evidence regarding the prevention of alcohol consumption of women of child bearing age in general, and particularly pregnant women, is not strong. Given this, the contributions arising from the analysis of expert opinion is even more important.

Although the initial scope of the FAR SEAS project was broadly multi-sectoral, the evidence found for FASD prevention was generally focused on health services and professionals, which represents a further limitation of the evidence review. However, the FAR SEAS Polish pilot showed promising results regarding the involvement of social services and social workers in the prevention and management of alcohol exposed pregnancies, which has been incorporated into the guidelines. The role of social care professionals should be further explored and assessed to synthesise new evidence around this sector, to inform guidelines and policy making, especially given that women in vulnerable social situations may be at higher risk of alcohol exposed pregnancies.

### Electronic supplementary material

Below is the link to the electronic supplementary material.

Supplementary Material 1

## Data Availability

Data supporting this article are available from the corresponding author on reasonable request.
