# Supplementary material for: Prevention of alcohol exposed pregnancies in Europe: the FAR SEAS guidelines
Source: BMC Pregnancy Childbirth. 2024 Apr 6;24:246. doi: 10.1186/s12884-024-06452-9 (PMC10998422; doi:10.1186/s12884-024-06452-9)
Supplement: Supplementary file 1 — Supplementary Material 1 [file 12884_2024_6452_MOESM1_ESM.pdf]

APPENDIX 1: TABLES AND FIGURES

Table 1: Grades of evidence based on the SIGN handbook

Level of evidence	Interpretation
**1^{++}**	High-quality meta-analyses, systematic reviews of RCTs, or RCTs with a very low risk of bias
**1^{+}**	Well-conducted meta-analyses, systematic reviews of RCTs, or RCTs with a low risk of bias
**1^{-}**	Meta-analyses, systematic reviews of RCTs with a high risk of bias
**2^{++}**	• High quality systematic reviews of case-control or cohort studies<br>• High quality case-control or cohort studies with a very low risk of confounding, bias or chance and a high probability that the relationship is causal
**2+**	Well-conducted case-control or cohort studies with a low risk of confounding, bias or chance and a moderate probability that the relationship is causal<br>Guidelines >70% AGREE score<br>Systematic review of cross-sectional studies
**2^{-}**	Case-control or cohort studies with a high risk of confounding, bias or chance and a significant risk that the relationship is not causal
**3**	Non-analytical studies (for example, case reports, case series)<br>Descriptive studies (cross-sectional study, qualitative, mixed methods, semi-structured interviews<br>Narrative reviews,<br>Guidelines <70% AGREE score
**4**	Expert opinion, formal consensus

Table 2: Guideline authors and collaborators

Role	Participant	Organization
Author	Carla Bruguera	Generalitat de Catalunya, Spain
Author	Katherin Rojas	Generalitat de Catalunya, Spain
Author and coordinator	Lidia Segura	Generalitat de Catalunya, Spain
Internal reviewer	Oscar Garcia-Algar	Fundació Clínic per la Recerca Biomèdica - Hospital Clínic, Spain
Internal reviewer	Marta Astals	Fundació Clínic per la Recerca Biomèdica - Hospital Clínic, Spain
Internal reviewer	Toni Gual	Fundació Clínic per la Recerca Biomèdica - Hospital Clínic, Spain
Internal reviewer	Silvia Matrai	Fundació Clínic per la Recerca Biomèdica - Hospital Clínic, Spain
Internal reviewer	Fleur Braddick	Fundació Clínic per la Recerca Biomèdica - Hospital Clínic, Spain
Internal reviewer	Katarzyna Okulicz	State Agency for the Prevention of Alcohol-Related Problems, Poland
Internal reviewer	Claudia Gandin	Istituto Superiore di Sanità, Italy
Internal reviewer	Emanuele Scafato	Istituto Superiore di Sanità, Italy

External advisor	Maria-Dolors Estrada	Agència de Qualitat I Avaluació Sanitàries de Catalunya, Spain
Internal reviewer and project manager	Joan Colom	Generalitat de Catalunya, Spain

Table 3: Members of expert group

Name and surname	Organization
Diane Black	The European Fetal Alcohol Spectrum Disorders Alliance
Lana Popova	Centre for Addiction and Mental Health, Canada
Lisa Schölin	Independent researcher, part of the UK FASD Research Collaboration, UK
Sylvia Roozen	Maastricht University, Neetherlands
Ilona Autti-Rämö	The council of choices in health care, the Ministry of Social Welfare and Health, Finland
Anna Klimkiewicz	Medical University of Warsaw, Poland
Javier Labad	Consorci Sanitari del Maresme, Spain
Lesley Smith	University of Hull
Thierry Maillard	SAF Ocean Indien
Lina Schwerg, Gela Becker	FASD-Fachzentrum Sonnenhof
Teresa Jadczak-Szumiło	Fundation Rodzina od A do Z
Berenice Doray	Resource Center Fetal Alcohol Spectrum Disorders (FASD) Reunion Island
Ann Boons	FAS Steunpunt
Martha Krijgsheld	Fasstichting Nederland (fasfoundation netherlands)
Steve Ondersma	Michigan State University, United States

Figure 1: Prisma flow chart

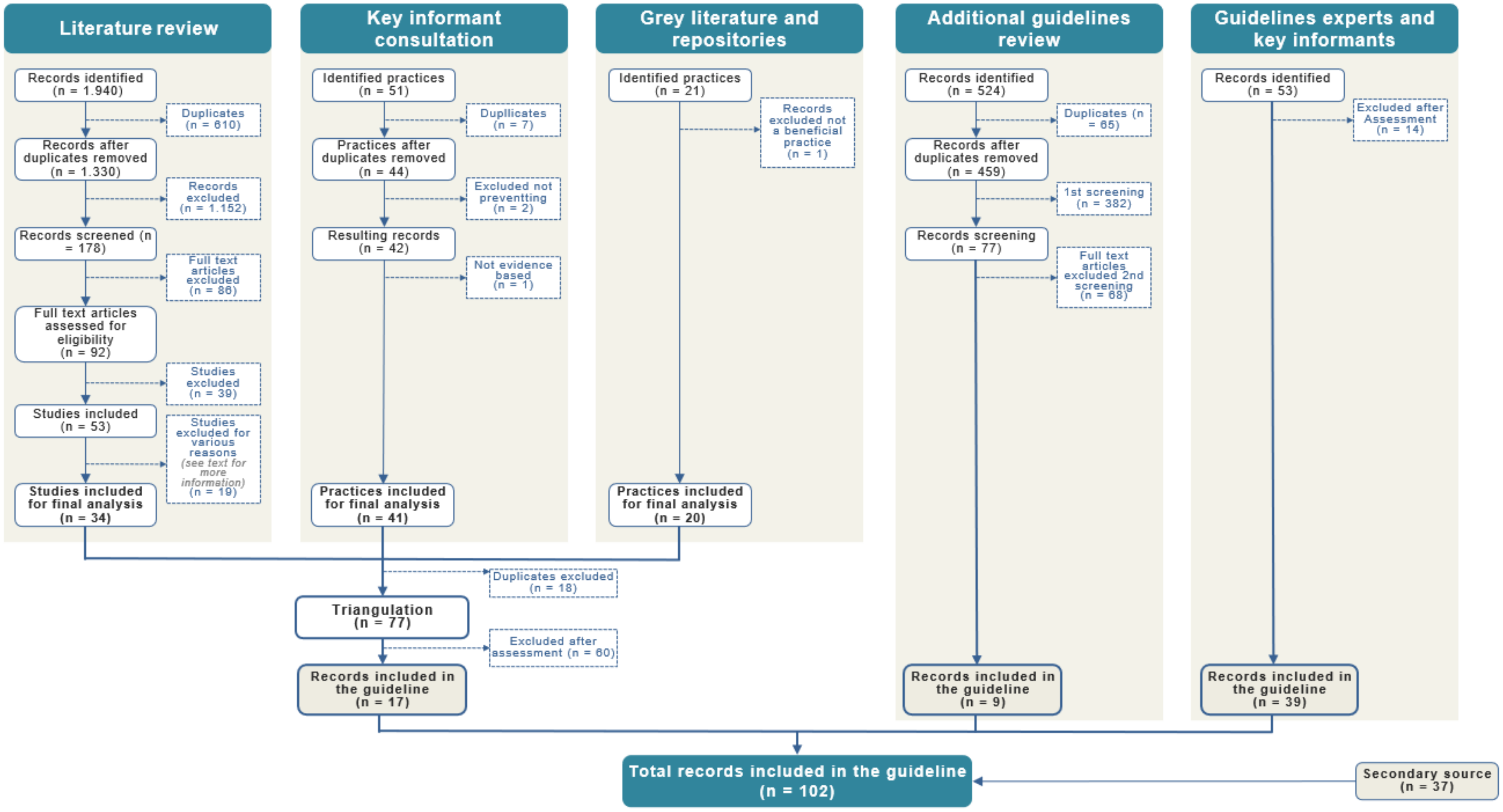

Table 4: 1) Organizational, strategic and policy changes required to properly address the needs of pregnant women and women of child-bearing age who are at risk of having, or who already have an alcohol-related problem

Question	Quality evidence	Recommendation	Strength	Benefits and harms
**1.1. Are there effective interventions to reduce stigmatization of women who use alcohol during pregnancy?**	No high-quality evidence was found regarding interventions that reduce stigma on women who drink during pregnancy. Overall SIGN grade: 3	1.1.1. Healthcare professionals should be trained in the knowledge and skills to address alcohol use in pregnancy (screening, treatment and referral) including:<br>• Understanding of the stigma around drinking during pregnancy and the complex factors that contribute to drinking during pregnancy.<br>• Language should be non-judgemental and supportive and avoid blaming and invoking guilt.	17 experts marked their level of agreement with the recommendation. The level of agreement of the group of experts with the recommendations was high.	• Benefits:<br>➢ Reduced stigma may increase likelihood of seeking help by women who consume alcohol during pregnancy or postpartum.<br>➢ Increase in the number of women receiving interventions for drinking during pregnancy leading to a reduction in FASD and other negative impacts of alcohol use on pregnancy outcomes.<br>• Harms:<br>➢ Healthcare providers may feel that this is adding
		1.1.2 Healthcare professionals should create a safe space for women to discuss their concerns regarding the	17 experts marked their level of agreement with the recommendation. The level of agreement	

		impacts of their drinking during pregnancy.	of the group of experts with the recommendations was high.	to an already heavy workload.
**1.2. Is there any legislation (legal measure) that has resulted in increased antenatal care among women who currently use or have used alcohol during pregnancy?**	No high-quality evidence was found regarding the impact of legislation on prenatal care among women who have used or currently use alcohol. Overall SIGN grade: 3	1.2.1 Legislation should aim to:<br>• Guarantee access to prevention, treatment and continuity of care.<br>• Respect patient autonomy, protect against discrimination and stigmatization and limit criminal prosecution.	16 experts marked their level of agreement with the recommendation. The level of agreement of the group of experts with the recommendation was high.	• Benefits:<br>➢ Supportive legal frameworks can increase the disclosure of alcohol use.<br>➢ Increase in prenatal care among women who use alcohol.<br>➢ Supportive legal frameworks could contribute to destigmatizing alcohol use during pregnancy.
**1.3. Which type of health and social policies should be introduced to reduce**	No high-quality evidence on which type of policies should be introduced to reduce alcohol	1.3.1 Policies must:<br>• Be comprehensive and multi-level and allow tailoring to the needs of women at differing levels of risk.	17 experts marked their level of agreement with the recommendation. The level of agreement of the group of experts	

**alcohol exposed pregnancies?**	exposed pregnancies was found. Overall SIGN grade: 4	• Support and coordinate, multidisciplinar networks of professionals at local level.	with the recommendation was high.	
		1.3.2 More research should be undertaken including the social contexts and determinants of alcohol use in pregnancy to inform policies to reduce alcohol exposed pregnancies.	17 experts marked their level of agreement with the recommendation. The level of agreement of the group of experts with the recommendation was high.	• Benefits:<br>➢ Universal screening policies and improved access to services and treatment.<br>➢ Better understanding of the prevalence and risk factors for alcohol use in pregnancy leading to better designed policies which address these.<br>➢ Better coordination and collaboration between health care services and between sectors.<br>➢ Less fear of punishment (i.e. losing custody of

				children) leading to higher likelihood of pregnant women disclosing alcohol use and their actual level of use.
**1.4. What organizational level factors increase delivery of preventive actions targeting the reduction of alcohol exposed pregnancies?**	No high-quality evidence was found regarding aspects at organization level that could increase delivery of preventive actions targeting the reduction of AEP. Overall SIGN grade: 3.	1.4.1. Organizational environments and resources must be enhanced to foster preventive activities:<br>• Clear, consistent and pragmatic guidelines, protocols and tools must be available for professionals.<br>• Structures and organizational policies must support continuity of care.<br>• Structures and policies should support multidisciplinary teams and interprofessional and service coordination and collaboration.	8 experts marked their level of agreement with the recommendation. The level of agreement of the group of experts with the recommendations was high.	• Benefits:<br>➢ Increase in women who use alcohol in pregnancy receiving appropriate interventions, support and treatment, and Reduction in alcohol exposed pregnancies due to:<br>○ Improved guidance for health professionals in addressing alcohol use in pregnancy.<br>○ Improved skills and knowledge among health professionals.

		• Professionals should be provided with relevant training and skills.<br>• Programmes should be evaluated and adapted to changing contexts and patient needs.		○ Increase in health professionals addressing alcohol use in pregnancy.<br>○ Women being at the centre of care and adapting of intervention to specific needs.<br>➢ Better coordination and collaboration between health professionals and between services.<br>➢ Improved continuity of care.
**1.5. What factors at the professional level increase delivery of preventive actions targeting the reduction of alcohol exposed pregnancies?**	No high-quality evidence was found regarding which changes at professional level could increase delivery of preventive actions targeting the reduction of alcohol	1.5.1. Barriers to health professionals addressing alcohol use in pregnancy should be identified and targeted with training and resources tailored to the local context.	8 experts marked their level of agreement with the recommendation. The level of agreement of the group of experts with the recommendations was high.	

	exposed pregnancies.. Overall SIGN grade: 3.			
		1.5.2. Key opinion leaders should be identified and recruited to promote acceptance and support for implementation of any activities, changes and interventions.	8 experts marked their level of agreement with the recommendation. The level of agreement of the group of experts with the recommendations was high.	• Benefits:<br>➢ Local champions can increase acceptance of new practices, structures and activities.<br>➢ Acceptance and engagement by practitioners is essential to the success of any change or new process.<br>➢ The preparation of healthcare providers is essential to be able to detect consumption, intervene and refer. When professionals are prepared, they are expected to be aware of prejudices and

				provide assurance to the patient that they can find support in the programs (Graves et al., 2020).<br><br>• Harms:<br><br>➢ Champions/key opinion leaders can be resistant to changing practice and suspicious of changes that may lead to increased workload and time pressures.
**1.6. What changes are needed to meet patients' needs for delivery of preventive actions?**	No high-quality evidence was found regarding changes needed to meet patients' needs for delivery of prevention actions. Overall SIGN grade: 3	1.6.1. Programmes should:<br><br>• Be holistic and consider each woman's specific circumstances and needs.<br><br>• Be implemented in an environment of safety and trust to	8 experts marked their level of agreement with the recommendation. The level of agreement of the group of experts with the recommendations was high.	

		promote engagement with support services or treatment programs.		
		1.6.2 Issues around child welfare and the potential involvement of child welfare agencies should be discussed honestly and openly, and with sensitivity.	8 experts marked their level of agreement with the recommendation. The level of agreement of the group of experts with the recommendations was high.	• Benefits:<br>➢ Comprehensive, integrated programs which address each woman's needs are more likely to support engagement with the intervention and have successful outcomes.<br>➢ When service providers take a supportive approach, it generates bonds and trust with the patient which in turn:<br>○ Increases the likelihood of them accepting help and adhering to treatment.

				○ Increases the likelihood of women discussing their alcohol use honestly.<br>• Harms:<br>➢ It is very complex for women to be able to trust service providers without feeling fear and guilt about the social and legal consequences, especially in relation to their children (Graves et al., 2020).

Table 5: 2) Strategies and best practices for promoting and raising awareness of the risks of drinking alcohol during pregnancy

Question	Quality evidence	Recommendation	Strength	Benefits and harms
**2.1. Which types of campaigns increase alcohol literacy and awareness of FASD**	No high-quality evidence was found regarding the type of campaigns which increase alcohol	2.1.1. Broad awareness raising and public health education campaigns using various media should be:<br>• Consistent with overall public health messaging.	8 experts marked their level of agreement with the recommendation. The level of agreement of the group of experts with	• Benefits:<br>➢ Increasing knowledge and awareness of the harm caused by alcohol

**among women of child-bearing age?**	literacy and awareness of FASD among women of child-bearing age. Overall SIGN grade: 4	• Comprehensively tested on different audiences and created in collaboration with relevant target groups to avoid stigmatization.	the recommendations was high.	consumption during pregnancy increases the likelihood of successful interventions, and reducing alcohol use in pregnancy (Crawford-Williams, Fielder, Mikocka-Walus & Esterman, 2015).<br><br>• Harms:<br>➢ Awareness does not always translate into changes in attitude or behaviour (Poole, Schmidt, Green & Hemsing, 2016).
**2.2. Which types of messages increase alcohol literacy and awareness of FASD**	No high-quality evidence was found regarding the types of messages that	2.2.1. Public health campaign messages targeting FASD prevention should combine information on the risks of alcohol use during pregnancy	8 experts marked their level of agreement with the recommendation. The level of agreement of	• Benefits:<br>➢ Increasing women's intentions to abstain from

**among women of child-bearing age?**	increase alcohol literacy and awareness of FASD among women of child-bearing age. Overall SIGN grade: 3.	combined with self-efficacy messages focusing on one's ability to adopt behaviour change taking care to avoid stigma and respect women's autonomy.	the group of experts with the recommendations was high.	alcohol during pregnancy (France et al., 2014).<br>• Harms:<br>➢ Certain messages could potentially produce defensive or maladaptive responses due to worry, guilt and shame (France et al., 2014).<br>➢ There is also the risk of stigmatising women who drink or have drunk during pregnancy and as a result reduce their chance to disclose or ask for help (Zizzo & Racine, 2017).<br>➢ Abstinence may be impossible for some women

				who struggle with addiction (Hocking, O'Callaghan & Reid, 2020; Zizzo & Racine, 2017).

Table 6: 3) Validated tools to screen alcohol use and maternal risk factors and further assess alcohol-related problems among pregnant women and women of child-bearing age in health and social care settings

Question	Quality evidence	Recommendation	Strength	Benefits and harms
**3.1. Which validated tools to screen and assess alcohol use in women of child-bearing age, especially pregnant women, have the highest sensitivity and specificity?**	No high-quality studies were identified. Given the risk of harm to the fetus, most guidelines recommended screening for alcohol use. Overall SIGN grade: 2+	3.1.1. Healthcare providers should ask all women of child-bearing age, especially those who are pregnant, about their alcohol use using a validated screening instrument.	8 experts marked their level of agreement with the recommendation. The level of agreement of the group of experts with the recommendations was high.	• Benefits:<br>➢ Universal screening is the first step to reduce alcohol consumption during pregnancy and in consequence improve maternal and child Health outcomes.

				• Harms:<br>➢ Universal screening might not be effective or cause harm if it is done in a judgemental way and there are potential legal or social consequences such as losing child for disclosing use.
**3.2. What psychosocial risk factors should be assessed when targeting alcohol exposed pregnancies?**	No high quality evidence was found regarding the psychosocial risk factors that should be assessed when targeting alcohol exposed pregnancies.<br>Overall SIGN grade: 3	3.2.1 Health professionals should screen all pregnant women for psychosocial risks given the correlation between alcohol use while pregnant and other complex problems that can have a negative impact on the woman, birth outcomes, and on the fetus and child.	8 experts marked their level of agreement with the recommendation. The level of agreement of the group of experts with the recommendations was high.	• Benefits:<br>➢ Some maternal risk factors can be predictors of alcohol consumption. Assessing them is crucial to help prevent alcohol-exposed pregnancies (19; National Institute for Health and Care Excellence, 2020; Center for Substance Abuse Prevention, 2014).<br>• Harms:

				➢ No high-quality evidence was identified examining the structure and content of the assessment.

Table 7: 4) Preventive interventions for pregnant women and women of child-bearing age at risk of having alcohol related problems

**Question**	**Quality evidence**	**Recommendation**	**Strength**	**Benefits and harms**
**4.1. Which interventions are most effective in preventing alcohol exposed pregnancies?**	No high-quality evidence was found regarding interventions that are effective in preventing alcohol exposed pregnancies. The level of evidence for most outcomes was low or very low.<br><br>However, the consulted guidelines	4.1.1 Health-care providers should offer tailored brief interventions to all women currently pregnant, or of child-bearing age and using alcohol or drugs, to prevent alcohol exposed pregnancies targeting alcohol use or contraception.	8 experts marked their level of agreement with the recommendation. The level of agreement of the group of experts with the recommendations was high.	• Benefits:<br><br>➢ Brief interventions have been associated with the following positive outcomes (WHO, 2014):<br><br>○ Reduced harmful consumption.<br><br>○ Reduced risk to the fetus.<br><br>○ Increased birthweight.

	supported a strong recommendation in favour of BIs because, although there was uncertainty about the degree of benefit, the potential benefit likely outweighs any potential harms.<br>Overall SIGN grade: 1+			○ Increase detection of harmful use and referral to treatment.<br>○ Improved general health of pregnant women.<br>○ Improved maternal psychological wellbeing.<br>○ Lower risk of fetotoxicity.<br>○ Improved perinatal outcomes (e.g. reduction in preterm births, increased overall birthweights, reduction in number of low-birthweight infants).<br>○ Reductions in congenital defects or anomalies.

				• Harms:<br>➢ Alcohol cessation and reduction could be difficult for some women (WHO, 2014) who could report they felt better drinking or that not drinking has created problems with their partner or friends if their interactions were based on alcohol consumption.
**4.2. Which components of Brief Interventions targeting alcohol use during pregnancy increase their effectiveness?**	No high-quality evidence was found regarding which components of BIs for alcohol use in pregnant women increase their effectiveness. Overall SIGN grade: 2+	4.2.1 Brief alcohol interventions should include:<br>• Assessment of readiness for change<br>• Advice on strategies for stopping use (specially on abstinence as a goal)<br>• Assistance eliciting ideas about change strategies	7 experts marked their level of agreement with the recommendation. The level of agreement of the group of experts with the recommendations was high.	• Benefits:<br>➢ Goal setting has been identified as an important behaviour change technique predicting adoption and maintenance of new health behaviours (Gilinsky, Swanson & Power, 2011).<br>• Harms:

		and/or referrals to support services.		➢ For some women these components will not be enough to stop drinking and will need more intense interventions (Gilinsky, Swanson & Power, 2011).
		4.2.2.Delivering synchronised interventions to partners (after asking for women's consent) is also recommended when feasible	8 experts marked their level of agreement with the recommendation. The level of agreement of the group of experts with the recommendations was high	

Table 8.1: 5) Treatment interventions for pregnant women and women of child-bearing age at risk of having alcohol related problems

Question	Quality evidence	Recommendation	Strength	Benefits and harms
**5.1. Which treatment interventions for pregnant women with an alcohol use disorder**	No high-quality evidence was found regarding the management of AUD	5.1.1 Alcohol use disorder during pregnancy should be addressed with individualized psychosocial support and short-	5 experts marked their level of agreement with the recommendation. The	Table 8.2

**improve maternal and child outcomes?**	during pregnancy.<br>Overall SIGN grade: 3	term use of a long-acting benzodiazepines if required (after assessment of withdrawal symptoms). However, if abstinence cannot be achieved, harm reduction strategies should be encouraged.	level of agreement was high.	
**5.2. Which strategies should be followed to provide continuity of care?**	No high-quality evidence was found regarding the management of AUD during pregnancy.<br>Overall SIGN grade: 2+	5.2.1. To support pregnant women undergoing treatment for alcohol use, continuity of care needs to be provided including:<br>• A coordinated multidisciplinary approach.<br>• Clear referral pathways should be established along with coordination between services and teams.	8 experts marked their level of agreement with the recommendation. The level of agreement of the group of experts with the recommendations was high	• Benefits:<br>➢ Pregnancy presents a unique opportunity to help support women to reduce and ideally cease alcohol use.<br>➢ Improved general health of pregnant women and psychological well-being.<br>➢ Reduction in risk to fetus.<br>➢ Improved perinatal outcomes (e.g. reduction in

		• An assertive follow-up post birth		preterm births, increased overall birthweights).<br>➢ Discussion with the paediatrician can help monitoring the child and a potential FASD diagnose (Graves et al., 2020).<br>• Harms:<br>➢ Referral for cessation intervention may induce time and economic burdens (Graves et al., 2020).

Table 8.2. Benefits and harms of recommendation 5.1.1*

Benefits	Harms
**Psychosocial interventions**	
➢ Pregnancy presents a unique opportunity to help support women to reduce and ideally cease alcohol and/or illicit substance use.	➢ Physical and mental symptoms associated with reduction or cessation.<br>➢ Possible development of depression or anxiety as a result of cessation or reduction.<br>➢ Possible risk of switching from one substance to another substance.

➢ Improved general health of pregnant women and psychological well-being.<br>➢ Reduction in risk to fetus.<br>➢ Improved perinatal outcomes (e.g. reduction in preterm births, increased overall birthweights)	Some people will experience a lack of bonding with the provider, lack of goal direction and monitoring, confrontation, criticism, and high emotional arousal and stigma.<br>➢ Stigmatization, risk of incarceration/loss of infant in punitive systems.<br>➢ Economic and time burdens imposed by need to attend interventions.<br>➢ Changes in behaviour can create conflicts at family level (partner/family), at work (employer over time) and commitment to the intervention.
**Pharmacological intervention for alcohol withdrawal**	
➢ Pregnancy presents a unique opportunity to support women to reduce and ideally cease alcohol and/or illicit substance use.<br>➢ Continued non-use following medication-assisted withdrawal is considered to be superior to usual care in terms of reduction in harmful consumption, reduction in risk to the fetus and improved perinatal outcomes.<br>➢ Improved general health of pregnant women and psychological well-being.	➢ The success of medication-assisted withdrawal during pregnancy is generally considered to be poor because of failure to complete detoxification or relapse.<br>➢ Stress symptoms following reduction or cessation and possible depression or anxiety.<br>➢ Increased risk of fetal distress, morbidity or mortality, including miscarriage and stillbirth.<br>➢ Possible risk of switching from one substance to another substance.<br>➢ Damage to relationships/loss of employment.<br>➢ BZDs cross the placenta and may bind to receptors in the developing fetal brain. BZDs use during the first trimester of pregnancy has been associated with an elevated risk of oral clefts and other malformations, with controversial results due to differences in methodological approaches. Maternal use of BZDs during late pregnancy (but sometimes earlier) was also associated with neonatal morbidity, an increased risk of preeclampsia and

	haemorrhage around delivery, preterm birth, low birth weight and height. A low Apgar score, an increased risk of hypoglycaemia and respiratory and neurological problems were also reported (19).
**Pharmacological intervention for maintenance of alcohol abstinence or reduction of use**	
➢ Helping maintain abstinence and reducing risk of alcohol exposed pregnancies.	➢ Unpleasant side effects of the pharmacological intervention.<br>➢ Possible development of depression or anxiety as a result of cessation or reduction.<br>➢ Possible risk of drug substitution.<br>➢ Possible increased risk of congenital defects and anomalies related to exposure to the pharmacological intervention.

* Information on this table comes from the WHO guidelines (18) except where noted otherwise

Table 9: **6) Social measures for pregnant women and women of child-bearing age at risk of having alcohol related problems**

Question	Quality evidence	Recommendation	Strength	Benefits and harms
**6.1. Which social interventions improve maternal and child outcomes?**	No high-quality evidence was found regarding social services or integrated models of care for women who have alcohol use	6.1.1. Women with alcohol use disorders should be provided with holistic support considering complex situations including poverty, lack of social support domestic violence among others.	17 experts marked their level of agreement with the recommendation. The level of agreement of the group of experts with the recommendations was high.	• Benefits:<br>➢ Holistic support which addresses not only alcohol use but also other related problems can increase the chances of abstinence.

	disorders.. Overall SIGN grade: 3.			➢ Comprehensive treatment approaches have the potential to improve outcomes for pregnant women with alcohol or other drug use problems and their babies (Pajulo et al., 2012; Breen, Awbery & Burns, 2014).<br><br>➢ When working in an integrated way between child and adult mental health, the mother's health problems are detected earlier, the effectiveness of the treatment increases and they last longer (Pajulo et al., 2012).<br><br>➢ Addressing the mother's health problems increases her own well-being, but also that of her child (Rutman,

				Hubberstey, Poole, Schmidt & Van Bibber, 2020).<br><br>• Harms:<br>➢ There are barriers to accessing services (Pajulo et al., 2012) and it involves resources and coordination efforts between multiple services (Rutman, Hubberstey, Poole, Schmidt & Van Bibber, 2020).<br>➢ Addressing mental and child health problems separately implies later and less effective detection (Pajulo et al., 2012).

Table 10: **7) Implementation, training and evaluation strategies for preventing activities**

**Question**	**Quality evidence**	**Recommendation**	**Strength**	**Benefits and harms**
**7.1. Which aspects of implementation increase delivery of preventive interventions targeting the reduction of alcohol exposed pregnancies?**	No high-quality evidence was found.	7.1.1. Preventive activities can be increased by implementing strategies such as:<br>• Training health professionals, with special attention to skills and familiarity with guidelines, resources and procedures.<br>• Improving clinical tools to screen, deliver brief interventions and refer to treatment.	7 experts marked their level of agreement with the recommendation. The level of agreement of the group of experts with the recommendations was high.	• Benefits:<br>➢ Raising health professionals' awareness of the risks of consuming during pregnancy, increases delivery of preventive activities (Lemola et al., 2020).<br>➢ Professionals training seems to increase delivery of preventive activities (Lemola et al., 2020; Sword et al., 2020; Breen, Awbery & Burns, 2014).

		• Mapping and collaborating with treatment services to which women will be referred.<br><br>• Naming a case coordinator involved in the woman's care to improve access to services.		➢ Assessing and addressing barriers could increase implementation effectiveness (Doherty et al., 2020).<br><br>➢ Early booking for care leads to improved pregnancy outcomes (National Collaborating Centre for Women's and Children's Health, 2010).<br><br>• Harms:<br><br>➢ The introduction of the guidelines alone will not result in increasing the preventive activities.
**7.2. Which aspects of professional training increase preventive actions targeting the**	No high-quality evidence was found. Overall SIGN grade: 4	7.2.1 Healthcare professionals should be given appropriate training and resources which provide them with the	8 experts marked their level of agreement with the recommendation. The level of agreement of the	• Benefits:<br>➢ Improving health professionals' skills and knowledge will result in

**reduction of alcohol exposed pregnancies?**		knowledge and skills needed to deliver effective preventive actions targeting the reduction of alcohol exposed pregnancies. This should include undergraduate and postgraduate training in the use of guidelines, screening tools, BIs and referral as well as appropriate communication techniques.	group of experts with the recommendation was high.	increased provision of information to users about the risks of alcohol use in pregnancy and consequently contribute to reducing the risk of alcohol exposed pregnancies (Sword et al., 2020).<br>• Harms:<br>➢ Without training providers, barriers will not be addressed, and the implementation of preventive activities might remain limited (Lemola et al., 2020; Sword et al., 2020; Breen, Awbery & Burns, 2014).
**7.3. How should preventive actions targeting the reduction of alcohol exposed**	No high-quality evidence was found. Recommendations made regarding this	7.3.1 To evaluate the implementation of preventive activities information should be collected on: the number of	8 experts marked their level of agreement with the recommendation. The level of agreement of the	• Benefits:<br>➢ Evaluation allows collecting information and

**pregnancies be evaluated and monitored?**	issue arise from two guidelines and a systematic review. In addition, two articles that did not fulfil criteria to be evaluated were included to answer this question. Overall SIGN grade: 3	staff trained in FASD prevention, patients screened, patients who have received an intervention, patients who have been referred to treatment services, and outcomes of the preventive activities such as abstinence, reduction of at-risk drinking and use of effective contraception.	group of experts with the recommendations was high.	monitoring the process allowing for improvements to be made which improve the quality of the service provided.<br>• Harms:<br>➢ If indicators of delivery of brief interventions are low because women screened report no alcohol consumption, professionals can think preventing alcohol use during pregnancy is a lower priority than other issues (Doi, Jepson & Cheyne, 2015).

Table 11: Records included

This table shows the list of documents used to generate the recommendations. In the table, in addition to finding the references, the type of document, the instrument to assess the quality of the evidence and the result obtained from it are identified.

To evaluate guidelines, the Agree II instrument was used (domain: methodological rigor), selecting those guidelines that reached a score greater than 70%.

For the systematic reviews (SRs) the AMSTAR instrument was used to assess methodological quality. SRs reaching medium (4 to 7) and high quality (8 to 11) using the AMSTAR 11-point checklist were included.

RCTs were assessed for risk of bias by evaluating five domains using the RoB2 instrument. Only those RCTs that did not show "high risk of bias" in any dimension were included. Finally, guidelines or documents were included which were considered of to address the questions, and at the suggestion of the experts.

Type	Reference	Quality assessment tool	Score	Reason for inclusion
Guideline	Thibaut F, Chagraoui A, Buckley L, Gressier F, Labad J, et al. (2019) Guidelines for the treatment of alcohol use disorders in pregnant women. World J Biol Psychiatry;20(1):17-50. Epub 2019 Jan 11. Erratum in: World J Biol Psychiatry. 2019 Apr 11;:1.	Agree II	71%	Score >70%
Guideline	Graves L, Carson G, Poole, N et al. (2020) Guideline No. 405: Screening and Counselling for Alcohol Consumption During Pregnancy J Obstet Gynaecol Can, 42, pp. 1162-1177	Agree II	81%	Score >70%
Other	Gelb K. & Rutman D. (2011). Substance Using Women with FASD and FASD Prevention: A Literature Review on Promising Approaches in Substance Use Treatment and Care for Women with FASD. Victoria, BC: University of Victoria.	Not rated	Not rated	Not rated
Guideline	National Institute for Health and Care Excellence. (2020). Antenatal and postnatal mental health: clinical management and service guidance. Retrieved on July 2, 2020, from https://www.nice.org.uk/guidance/cg192/evidence/full-guideline-pdf-4840896925	Agree II	98%	Score >70%
Guideline	National Collaborating Centre for Women's and Children's Health (UK). (2010) Pregnancy and Complex Social Factors: A Model for Service Provision for Pregnant Women with Complex Social Factors. London: RCOG Press.	Agree II	98%	Score >70%

Guideline	WHO (2014), Guidelines for the identification and management of substance use and substance use disorders in pregnancy. Retrieved on July 2, 2020, from https://apps.who.int/iris/bitstream/handle/10665/107130/9789241548731_eng.pdf;jsessionid=A6556244BD91CF2E4CAE55A07ACA33E7?sequence=1	Agree II	100%	Score >70%
SR	Fergie L, Campbell KA, Coleman-Haynes T, Ussher M, Cooper S, Coleman T. (2019) Identifying Effective Behavior Change Techniques for Alcohol and Illicit Substance Use During Pregnancy: A Systematic Review. Ann Behav Med. Jul 17;53(8):769-781. doi: 10.1093/abm/kay085.	Amstar	8	>4 points
RCT	van der Wulp NY, Hoving C, Eijmael,K, Candel MJ, van Dalen W, & De Vries H. (2014). Reducing alcohol use during pregnancy via health counseling by midwives and internet-based computer-tailored feedback: a cluster randomized trial. Journal of medical Internet research, 16(12), e274. https://doi.org/10.2196/jmir.3493	RoB2	No high risk domain	Not high risk
RCT	Bortes C, Geidne,S. and Eriksson C. (2015) Preventing Alcohol Consumption during Pregnancy: A Randomized Controlled Trial. Health, 7, 289-299. http://dx.doi.org/10.4236/health.2015.73033	RoB2	No high risk domain	Not high risk
RCT	Velasquez MM, von Sternberg KL, Floyd RL, Parrish D, Kowalchuk A, et al. (2017). Preventing Alcohol and Tobacco Exposed Pregnancies: CHOICES Plus in Primary Care. American journal of preventive medicine, 53(1), 85–95. https://doi.org/10.1016/j.amepre.2017.02.012	RoB2	No high risk domain	Not high risk
RCT	Dresser J, Starling R, Woodall WG, Stanghetta P, & May PA. (2011). Field trial of alcohol-server training for prevention of fetal alcohol syndrome. Journal of studies on alcohol and drugs, 72(3), 490–496. https://doi.org/10.15288/jsad.2011.72.490	RoB2	No high risk domain	Not high risk
RCT	Ingersoll K, Frederick C, MacDonnell,K, Ritterband L, Lord H, Jones,B, & Truwit L. (2018). A Pilot RCT of an Internet Intervention to Reduce the Risk of Alcohol-Exposed Pregnancy. Alcoholism, clinical and experimental research, 42(6), 1132–1144. https://doi.org/10.1111/acer.13635	RoB2	No high risk domain	Not high risk
RCT	Evans WD, Wallace Bihm J, Szekely D, Nielsen P, Murray E, et al. (2014). Initial outcomes from a 4-week follow-up study of the Text4baby program in the military women's population: randomized controlled trial. Journal of medical Internet research, 16(5), e131. https://doi.org/10.2196/jmir.3297	RoB2	No high risk domain	Not high risk
RCT	Tzilos Wernette G, Plegue M, Kahler CW, Sen A, & Zlotnick,C. (2018). A Pilot Randomized Controlled Trial of a Computer-Delivered Brief Intervention for Substance Use and Risky Sex During Pregnancy. Journal of women's health (2002), 27(1), 83–92. https://doi.org/10.1089/jwh.2017.6408	RoB2	No high risk domain	Not high risk

SR	Gilinsky A, Swanson V & Power KG (2011). Interventions delivered during antenatal care to reduce alcohol consumption during pregnancy: A systematic review, Addiction Research and Theory, 19 (3), 235-250.	Amstar	7	>4 points
SR	Roozen S, Peters GY, Kok G, Townend D, Nijhuis J, Koek G, & Curfs, L. (2018). Systematic literature review on which maternal alcohol behaviours are related to fetal alcohol spectrum disorders (FASD). BMJ open, 8(12), e022578. https://doi.org/10.1136/bmjopen-2018-022578	Amstar	8	>4 points
Other	Högberg H, Spak F, & Larsson M. (2015). Dialogue between Midwives and Parents-to-Be about Alcohol, from a Life Cycle Perspective - An Intervention Study. Creative Education, 6, 489-500. http://dx.doi.org/10.4236/ce.2015.65049	Not rated	Not rated	Not rated
Other	Brems C, Boschma-Wynn RV, Dewane SL, Edwards AE, Robinson RV. (2010) Training needs of healthcare providers related to Centers for Disease Control and Prevention core competencies for fetal alcohol spectrum disorders. J Popul Ther Clin Pharmacol. 2010 Fall;17(3):e405-17. Epub 2010 Nov 1.	Not rated	Not rated	Not rated
Other	Schölin, L. (2016) Prevention of harm caused by alcohol exposure in pregnancy. Rapid review and case studies from Member States. Geneva: World Health Organization Regional Office for Europe. Accessible at: https://www.euro.who.int/__data/assets/pdf_file/0005/318074/Prevention-harm-caused-alcohol-exposure-pregnancy.pdf	Not rated	Not rated	Not rated
Other	Pajulo M, Pyykkönen N, Kalland M, Sinkkonen J, Helenius H, et al. (2012). Substance-abusing mothers in residential treatment with their babies: importance of pre- and postnatal maternal reflective functioning. Infant mental health journal, 33(1), 70–81. https://doi.org/10.1002/imhj.20342	Not rated	Not rated	Not rated
Other	O'Brien PL. (2014). Performance measurement: a proposal to increase use of SBIRT and decrease alcohol consumption during pregnancy. Maternal and child health journal, 18(1), 1–9. https://doi.org/10.1007/s10995-013-1257-2	Not rated	Not rated	Not rated
Other	Hocking M, O'Callaghan F, & Reid, N. (2020). Women's experiences of messages relating to alcohol consumption, received during their first antenatal care visit: An interpretative phenomenological analysis. Women and birth : journal of the Australian College of Midwives, 33(2), e122–e128. https://doi.org/10.1016/j.wombi.2019.02.002	Not rated	Not rated	Not rated
Other	McBride N. (2014). Alcohol use during pregnancy: considerations for Australian policy. Social work in public health, 29(6), 540–548. https://doi.org/10.1080/19371918.2014.890150	Not rated	Not rated	Not rated

Other	Poole, N, & Greaves, L. (2013). Alcohol use during pregnancy in Canada: how policy moments can create opportunities for promoting women's health. Canadian journal of public health = Revue canadienne de sante publique, 104(2), e170–e172. https://doi.org/10.1007/BF03405683	Not rated	Not rated	Not rated
Other	Zizzo, N, Racine, E. (2017) Ethical challenges in FASD prevention: Scientific uncertainty, stigma, and respect for women's autonomy. Can J Public Health 108, 414–417. https://doi.org/10.17269/CJPH.108.6048	Not rated	Not rated	Not rated
Other	Driscoll DL, Barnes VR, Johnston JM, Windsor R, Ray R, A (2018) Formative Evaluation of Two FASD Prevention Communication Strategies, Alcohol and Alcoholism, Volume 53, Issue 4, Pages 461–469, https://doi.org/10.1093/alcalc/agx122	Not rated	Not rated	Not rated
Other	Thomas, G, Gonneau, G, Poole, N, & Cook, J. (2014). The effectiveness of alcohol warning labels in the prevention of Fetal Alcohol Spectrum Disorder: A brief review. The International Journal of Alcohol and Drug Research, 3(1), 91-103. https://doi.org/10.7895/ijadr.v3i1.126	Not rated	Not rated	Not rated
SR	Symons, M, Pedruzzi, R. A, Bruce, K, & Milne, E. (2018). A systematic review of prevention interventions to reduce prenatal alcohol exposure and fetal alcohol spectrum disorder in indigenous communities. BMC public health, 18(1), 1227. https://doi.org/10.1186/s12889-018-6139-5	Amstar	9	>4 points
Other	Poole, N, Schmidt, R. A, Green, C, & Hemsing, N. (2016). Prevention of Fetal Alcohol Spectrum Disorder: Current Canadian Efforts and Analysis of Gaps. Substance abuse : research and treatment, 10(Suppl 1), 1–11. https://doi.org/10.4137/SART.S34545	Not rated	Not rated	Not rated
Other	Corrigan, P. W, Shah, B. B, Lara, J. L, Mitchell, K. T, Simmes, D, & Jones, K. L. (2018). Addressing the public health concerns of Fetal Alcohol Spectrum Disorder: Impact of stigma and health literacy. Drug and alcohol dependence, 185, 266–270. https://doi.org/10.1016/j.drugalcdep.2017.12.027	Not rated	Not rated	Not rated
Other	Corrigan, P. W, Lara, J. L, Shah, B. B, Mitchell, K. T, Simmes, D, & Jones, K. L. (2017). The Public Stigma of Birth Mothers of Children with Fetal Alcohol Spectrum Disorders. Alcoholism, clinical and experimental research, 41(6), 1166–1173. https://doi.org/10.1111/acer.13381	Not rated	Not rated	Not rated
Other	Bazzo, S, Battistella, G, Riscica, P, Moino, G, Marini, F, et al. (2015). Evaluation of a Multilevel and Integrated Program to Raise Awareness of the Harmful Effects of Prenatal Alcohol Exposure in a Local Community. Alcohol and alcoholism (Oxford, Oxfordshire), 50(6), 708–715. https://doi.org/10.1093/alcalc/agv051	Not rated	Not rated	Not rated
Other	Pichini, S, Marchei, E, Vagnarelli, F, Tarani, L, Raimondi, F, et al. (2012). Assessment of prenatal exposure to ethanol by meconium analysis: results of an Italian multicenter study. Alcoholism, clinical and experimental research, 36(3), 417–424. https://doi.org/10.1111/j.1530-0277.2011.01647.x	Not rated	Not rated	Not rated

Other	France, K. E, Donovan, R. J, Bower, C, Elliott, E. J, Payne, J. et al. (2014). Messages that increase women's intentions to abstain from alcohol during pregnancy: results from quantitative testing of advertising concepts. BMC public health, 14, 30. https://doi.org/10.1186/1471-2458-14-30	Not rated	Not rated	Not rated
Other	Lemola, S, Gkiouleka, A, Urfer-Maurer, N. et al. (2020) Midwives' engagement in smoking- and alcohol-prevention in prenatal care before and after the introduction of practice guidelines in Switzerland: comparison of survey findings from 2008 and 2018. BMC Pregnancy Childbirth 20, 31. https://doi.org/10.1186/s12884-019-2706-8	Not rated	Not rated	Not rated
Other	Doherty, E, Kingsland, M, Wiggers, J, Anderson, A. E, Elliott, E. J, et al. (2020). Barriers to the implementation of clinical guidelines for maternal alcohol consumption in antenatal services: A survey using the theoretical domains framework. Health promotion journal of Australia : official journal of Australian Association of Health Promotion Professionals, 31(1), 133–139. https://doi.org/10.1002/hpja.258	Not rated	Not rated	Not rated
Other	Sword W, Green C, Akhtar-Danesh N, et al. (2020) Screening and Intervention Practices for Alcohol Use by Pregnant Women and Women of Childbearing Age: Results of a Canadian Survey. Journal of Obstetrics and Gynaecology Canada : JOGC = Journal D'obstetrique et Gynecologie du Canada : JOGC. Sep;42(9):1121-1128.	Not rated	Not rated	Not rated
Guideline	Center for Substance Abuse Prevention (US). (2014). Addressing Fetal Alcohol Spectrum Disorders (FASD). Substance Abuse and Mental Health Services Administration (US).	Agree II	25%	Expert Tip
Guideline	Centers for Disease Control and Prevention. (2014) Planning and implementing screening and brief intervention for risky alcohol use. Atlanta: Centers for Disease Control and Prevention, National Center on Birth Defects and Developmental Disabilities.	Agree II	17%	Expert Tip
Guideline	Breen, C, Awbery, E. and Burns, L. (2014), Supporting Pregnant Women who use Alcohol or other Drugs: A Guide for Primary Health Care Professionals. Retrieved on August 20, 2020, from https://ndarc.med.unsw.edu.au/sites/default/files/ndarc/resources/Supporting%20Pregnant%20Women%20who%20use%20Alcohol%20or%20Other%20Drugs%20-%20A%20review%20of%20the%20evidence.pdf	Agree II	23%	Expert Tip
RCT	Montag A, Dusek M, Mazzetti A, Nelson L, Ortega M, Campillo A, Calac D, Nyquist C, Chambers C. (2014). Preventing fetal alcohol spectrum disorders in a native American population. Alcoholism: clinical and experimental research; 38.	RoB2	No high risk domain	Not high risk
RCT	Martino, S, Ondersma, S. J, Forray, A, Olmstead, T. A, Gilstad-Hayden, K, et al. (2018). A randomized controlled trial of screening and brief interventions for substance misuse in reproductive health. American journal of obstetrics and gynecology, 218(3), 322.e1–322.e12. https://doi.org/10.1016/j.ajog.2017.12.005	RoB2	No high risk domain	Not high risk

Other	Forray A, Martino S, Gilstad-Hayden K, et al. (2019). Assessment of an electronic and clinician-delivered brief intervention on cigarette, alcohol and illicit drug use among women in a reproductive healthcare clinic. Addict Behav. 96:156-163. doi:10.1016/j.addbeh.2019.05.007	Not rated	Not rated	Not rated
Other	Olmstead, T. A, Yonkers, K. A, Ondersma, S. J, Forray, A, Gilstad-Hayden, K, et al. (2019). Cost-effectiveness of electronic- and clinician-delivered screening, brief intervention and referral to treatment for women in reproductive health centers. Addiction (Abingdon, England), 114(9), 1659–1669. https://doi.org/10.1111/add.14668	Not rated	Not rated	Not rated
Other	Marcellus L, MacKinnon K, Benoit C, Phillips R, & Stengel C. (2015). Reenvisioning Success for Programs Supporting Pregnant Women With Problematic Substance Use. Qualitative Health Research, 25(4), 500–512. https://doi.org/10.1177/1049732314551058	Not rated	Not rated	Not rated
Guideline	NSW Ministry of Health (2014) Clinical guidelines for the Management of Substance Use During Pregnancy Birth and the Postnatal Period. Retrieved on August 20, 2020, from https://www.health.nsw.gov.au/aod/professionals/Pages/substance-use-during-pregnancy-guidelines.aspx	Agree II	4%	Expert Tip
Other	Edwards A, Kelsey B, Pierce-Bulger M, Rawlins S, Ruhl C, et al. (2020). Applying Ethical Principles When Discussing Alcohol Use During Pregnancy. Journal of midwifery & women's health, 10.1111/jmwh.13159. Advance online publication. https://doi.org/10.1111/jmwh.13159	Not rated	Not rated	Not rated
Other	Roozen S, Stutterheim SE, Bos E, Kok, G, & Curfs LM. (2020) Understanding the Social Stigma of Fetal Alcohol Spectrum Disorders: From Theory to Interventions. Foundations of Science:1-19.	Not rated	Not rated	Not rated
Other	Bakhireva LN, Wilsnack SC, Kristjanson A, Yevtushok L, Onishenko S, et al. (2011). Paternal drinking, intimate relationship quality, and alcohol consumption in pregnant Ukrainian women. Journal of studies on alcohol and drugs, 72(4), 536–544. https://doi.org/10.15288/jsad.2011.72.536	Not rated	Not rated	Not rated
Other	Czeizel AE, Czeizel B, & Vereczkey A. (2013). The participation of prospective fathers in preconception care. Clinical medicine insights. Reproductive health, 7, 1–9. https://doi.org/10.4137/CMRH.S10930	Not rated	Not rated	Not rated
Other	Frey KA, Engle R, & Noble B. (2012). Preconception healthcare: What do men know and believe? Journal of Men's Health, 9(1), 25-35. https://doi.org/10.1016/j.jomh.2011.11.001	Not rated	Not rated	Not rated
Other	Milne, E, Greenop, K. R, Scott, R. J, de Klerk, N. H, Bower, C, et al. (2013). Parental alcohol consumption and risk of childhood acute lymphoblastic leukemia and brain tumors. Cancer causes & control : CCC, 24(2), 391–402. https://doi.org/10.1007/s10552-012-0125-5	Not rated	Not rated	Not rated

Other	Klonoff-Cohen, H, Lam-Kruglick, P, & Gonzalez, C. (2003). Effects of maternal and paternal alcohol consumption on the success rates of in vitro fertilization and gamete intrafallopian transfer. Fertility and Sterility, 79(2), 330–339. https://doi.org/10.1016/S0015-0282(02)04582-X	Not rated	Not rated	Not rated
Other	van der Wulp, N. Y, Hoving, C, & de Vries, H. (2015). Partner's influences and other correlates of prenatal alcohol use. Maternal and child health journal, 19(4), 908–916. https://doi.org/10.1007/s10995-014-1592-y	Not rated	Not rated	Not rated
Other	Drabble, L, Thomas, S, O'Connor, L, & Roberts, S. C. (2014). State Responses to Alcohol Use and Pregnancy: Findings From the Alcohol Policy Information System (APIS). Journal of social work practice in the addictions, 14(2), 191–206. https://doi.org/10.1080/1533256X.2014.900409	Not rated	Not rated	Not rated
Other	Roberts, S, Mericle, A. A, Subbaraman, M. S, Thomas, S, Treffers, R. D, et al. (2019). State Policies Targeting Alcohol Use during Pregnancy and Alcohol Use among Pregnant Women 1985-2016: Evidence from the Behavioral Risk Factor Surveillance System. Women's health issues : official publication of the Jacobs Institute of Women's Health, 29(3), 213–221. https://doi.org/10.1016/j.whi.2019.02.001	Not rated	Not rated	Not rated
Other	Crawford-Williams F, Fielder A, Mikocka-Walus A, & Esterman A. (2015). A critical review of public health interventions aimed at reducing alcohol consumption and/or increasing knowledge among pregnant women. Drug and alcohol review, 34(2), 154–161. https://doi.org/10.1111/dar.12152	Not rated	Not rated	Not rated
Other	Ethen MK, Ramadhani TA, Scheuerle AE, Canfield MA, Wyszynski DF, et al. National Birth Defects Prevention Study (2009). Alcohol consumption by women before and during pregnancy. Maternal and child health journal, 13(2), 274–285. https://doi.org/10.1007/s10995-008-0328-2	Not rated	Not rated	Not rated
Other	Oni HT, Buultjens M, Abdel-Latif ME, & Islam MM. (2019). Barriers to screening pregnant women for alcohol or other drugs: A narrative synthesis. Women and birth : journal of the Australian College of Midwives, 32(6), 479–486. https://doi.org/10.1016/j.wombi.2018.11.009	Not rated	Not rated	Not rated
Other	Oni HT, Buultjens M, Blandthorn J, Davis D, Abdel-Latif M, et al. (2020). Barriers and facilitators in antenatal settings to screening and referral of pregnant women who use alcohol or other drugs: A qualitative study of midwives' experience. Midwifery, 81, 102595. https://doi.org/10.1016/j.midw.2019.102595	Not rated	Not rated	Not rated
Other	Rutman, D, Hubberstey, C, Poole, N. et al. (2020) Multi-service prevention programs for pregnant and parenting women with substance use and multiple vulnerabilities: Program structure and clients' perspectives on wraparound programming. BMC Pregnancy Childbirth 20, 441. https://doi.org/10.1186/s12884-020-03109-1	Not rated	Not rated	Not rated

Other	Weile L, Wu C, Hegaard HK, Kesmodel US, Henriksen, et al. (2020). Identification of Alcohol Risk Drinking Behaviour in Pregnancy Using a Web-Based Questionnaire: Large-Scale Implementation in Antenatal Care. Alcohol and alcoholism (Oxford, Oxfordshire), 55(2), 225–232. https://doi.org/10.1093/alcalc/agz100	Not rated	Not rated	Not rated
Other	Tenkku LE, Mengel MB, Nicholson RA, Hile MG, Morris DS, et al. (2011). A web-based intervention to reduce alcohol-exposed pregnancies in the community. Health education & behavior : the official publication of the Society for Public Health Education, 38(6), 563–573. https://doi.org/10.1177/1090198110385773	Not rated	Not rated	Not rated
Other	Pollick SA, Beatty JR, Sokol J, Strickler, RC, Chang G, et al. (2015). Acceptability of a computerized brief intervention for alcohol among abstinent but at-risk pregnant women. Substance abuse, 36(1), 13–20. https://doi.org/10.1080/08897077.2013.857631	Not rated	Not rated	Not rated
SR	Gilinsky A, Swanson V & Power KG. (2011) Interventions delivered during antenatal care to reduce alcohol consumption during pregnancy: A systematic review. Addiction Research and Theory, 19 (3), pp. 235-250. https://doi.org/10.3109/16066359.2010.507894	Amstar	7	>4 points
RCT	Ingersoll KS, Ceperich SD, Hettema JE, Farrell-Carnahan L, & Penberthy JK. (2013). Preconceptional motivational interviewing interventions to reduce alcohol-exposed pregnancy risk. Journal of substance abuse treatment, 44(4), 407–416. https://doi.org/10.1016/j.jsat.2012.10.001	RoB2	No high risk domain	Not high risk
RCT	Ceperich S, Ingersoll K. (2011) Motivational interviewing + feedback intervention to reduce alcohol-exposed pregnancy risk among college binge drinkers: determinants and patterns of response. J Behav Med;34:381–95.	RoB2	No high risk domain	Not high risk
RCT	Floyd RL, Sobell M, Velasquez MM, et al. (2007) Preventing alcohol-exposed pregnancies: a randomized controlled trial. Am J Prev Med;32:1–10.	RoB2	No high risk domain	Not high risk
RCT	Rendall-Mkosi K, Morojele N, London L, Moodley S, Singh C, Girdler-Brown B. 2013. A randomized controlled trial of motivational interviewing to prevent risk for an alcoholexposed pregnancy in the Western Cape, South Africa. Addiction. 108:725–732	RoB2	No high risk domain	Not high risk
Other	Ondersma SJ, Chase SK, Svikis DS, & Schuster CR. (2005). Computer-based brief motivational intervention for perinatal drug use. Journal of substance abuse treatment, 28(4), 305–312. https://doi.org/10.1016/j.jsat.2005.02.004	Not rated	Not rated	Not rated

RCT	Tzilos GK, Sokol RJ, & Ondersma SJ. (2011). A randomized phase I trial of a brief computer-delivered intervention for alcohol use during pregnancy. Journal of women's health (2002), 20(10), 1517–1524. https://doi.org/10.1089/jwh.2011.2732	RoB2	No high risk domain	Not high risk
RCT	Ondersma SJ, Beatty JR, Svikis DS, Strickler RC, Tzilos GK, et al. (2015). Computer-Delivered Screening and Brief Intervention for Alcohol Use in Pregnancy: A Pilot Randomized Trial. Alcoholism, clinical and experimental research, 39(7), 1219–1226. https://doi.org/10.1111/acer.12747	RoB2	No high risk domain	Not high risk
Other	Hanson JD, & Pourier S. (2015). The Oglala Sioux Tribe CHOICES Program: Modifying an Existing Alcohol-Exposed Pregnancy Intervention for Use in an American Indian Community. International journal of environmental research and public health, 13(1), ijerph13010001. https://doi.org/10.3390/ijerph13010001	Not rated	Not rated	Not rated
Other	Wolfson, L, Poole, N, Morton Ninomiya, M, Rutman, D, Letendre, S, e (2019). Collaborative Action on Fetal Alcohol Spectrum Disorder Prevention: Principles for Enacting the Truth and Reconciliation Commission Call to Action #33. International journal of environmental research and public health, 16(9), 1589. https://doi.org/10.3390/ijerph16091589	Not rated	Not rated	Not rated
SR	Burns E, Gray R, & Smith LA. (2010). Brief screening questionnaires to identify problem drinking during pregnancy: a systematic review. Addiction (Abingdon, England), 105(4), 601–614. https://doi.org/10.1111/j.1360-0443.2009.02842.x	Amstar	10	>4 points
SR	Henderson J, Gra, R, & Brocklehurst P. (2007). Systematic review of effects of low-moderate prenatal alcohol exposure on pregnancy outcome. BJOG : an international journal of obstetrics and gynaecology, 114(3), 243–252. https://doi.org/10.1111/j.1471-0528.2006.01163.x	Amstar	8	>4 points

Table 12: Files evaluation template

Evaluation interpretation

- Randomised Control Trials to be analysed by using RoB2, the revised Cochrane risk-of-bias tool for RCTs. Studies with one or more high-risk domain will be excluded.
- Systematic reviews to be assessed using AMSTAR. Reviews scoring less than 4 items will be excluded.
- Guidelines to be valuated by using the section Rigour of Development criteria of the AGREE II instrument (13). Guidelines scoring less than 70% will be excluded.

**ID**		
**Source type (Systematic review, RCT, guidelines)**		
**Reference**		
**Agree II - Rigour of development criteria: https://www.agreetrust.org/wp-content/uploads/2017/12/AGREE-II-Users-Manual-and-23-item-Instrument-2009-Update-2017.pdf**	**7. SEARCH METHODS (1-7)**	
	**8. EVIDENCE SELECTION CRITERIA (1-7)**	
	**9. STRENGTHS & LIMITATIONS OF THE EVIDENCE (1-7)**	
	**10. FORMULATION OF RECOMMENDATIONS (1-7)**	
	**11. CONSIDERATION OF BENEFITS AND HARMS (1-7)**	
	**12. LINK BETWEEN RECOMMENDATIONS AND EVIDENCE (1-7)**	
	**13. EXTERNAL REVIEW (1-7)**	
	**14. UPDATING PROCEDURE (1-7)**	
	**Score (%)**	
**Calidad metodológica de revisiones sistemáticas AMSTAR**	**1**	
	**2**	
	**3**	
	**4**	
	**5**	
	**6**	
	**7**	
	**8**	
	**9**	
	**10**	
	**11**	
	**Score over 11**	
**ROB 2: Domain 1**	**1.1**	
	**1.2**	
	**1.3**	

	Judgement D1	
ROB 2: Domain 2	2.1	
	2.2	
	2.3	
	2.4	
	2.5	
	2.6	
	2.7	
	Judgement D2	
ROB 2: Domain 3	3.1	
	3.2	
	3.3	
	3.4	
	Judgement D3	
ROB 2: Domain 4	4.1	
	4.2	
	4.3	
	4.4	
	4.5	
	Judgement D4	
ROB 2: Domain 5	5.1	
	5.2	
	5.3	
	Judgement D5	
